# Correct recognition and continuum belief of mental disorders in a nursing student population

**DOI:** 10.1186/s12888-017-1447-3

**Published:** 2017-08-07

**Authors:** Lee Seng Esmond Seow, Boon Yiang Chua, Huiting Xie, Jia Wang, Hui Lin Ong, Edimansyah Abdin, Siow Ann Chong, Mythily Subramaniam

**Affiliations:** 1 Research Division, Institute of Mental Health, Singapore, Singapore; 2 Nursing Administration, Institute of Mental Health, Singapore, Singapore

## Abstract

**Background:**

The current study aimed to explore the correct recognition of mental disorders across dementia, alcohol abuse, obsessive compulsive disorder (OCD), schizophrenia and depression, along with its correlates in a nursing student population. The belief in a continuum of symptoms from mental health to mental illness and its relationship with the non-identification of mental illness was also explored.

**Methods:**

Five hundred students from four nursing institutions in Singapore participated in this cross-sectional online study. Respondents were randomly assigned to a vignette describing one of the five mental disorders before being asked to identify what the person in the vignette is suffering from. Continuum belief was assessed by rating their agreeableness with the following statement: “Sometimes we all behave like X. It is just a question of how severe or obvious this condition is”.

**Results:**

OCD had the highest correct recognition rate (86%), followed by depression (85%), dementia (77%), alcohol abuse (58%) and schizophrenia (46%). For continuum belief, the percentage of respondents who endorsed symptom continuity were 70% for depression, 61% for OCD, 58% for alcohol abuse, 56% for dementia and 46% for schizophrenia**.** Of concern, we found stronger continuum belief to be associated with the non-identification of mental illness after controlling for covariates.

**Conclusions:**

There is a need to improve mental health literacy among nursing students. Almost a quarter of the respondents identified excessive alcohol drinking as depression, even though there was no indication of any mood symptom in the vignette on alcohol abuse. Further education and training in schizophrenia may need to be conducted. Healthcare trainees should also be made aware on the possible influence of belief in symptom continuity on one’s tendency to under-attribute mental health symptoms as a mental illness.

## Background

Mental health literacy (MHL) is the knowledge and beliefs about mental disorders that influence recognition, treatment and prevention. The US Institute of Medicine (IoM) defined health literacy as “the degree to which individuals have capacity to obtain, process and understand basic health information and services needed to make appropriate health decisions” [1]. Close to 50% of the US population reported lifetime prevalence of at least one mental disorder while nearly 30% reported at least one 12-month mental disorder [2]. The Global Burden of Disease Study (GBD 2010) estimated that mental, neurological and substance use disorders accounted for 10.4% of global disability-adjusted life years (DALYs), 2.3% of global years of life (YLLs) lost to premature mortality and 28.5% of global years lived with disability (YLDs), making them the leading cause of YLDs [3]. With the high prevalence and the significant proportion of disease burden due to mental illnesses, the concept of mental health literacy thus becomes crucial as knowledge about mental health diseases serves as a prerequisite for early recognition and intervention in mental disorders.

Studies have shown that many members of the public cannot understand the meaning of psychiatric terms or correctly recognize mental health problems [4, 5]. Physicians and healthcare professionals therefore play an important role in detecting mental disorders in the society as they are the individuals whom the public will first seek help and treatment from. However, mental health problems and existing mental disorders often remain undiagnosed in the primary care setting [6, 7]. In one study, only 23% of a surgical patient population was accurately identified by the surgeons and nurses for their substance abuse, with the lack of time and knowledge being the reasons for this low recognition rate [8]. In addition, patients may often present with somatic complaints that mimic those of a diagnosed medical condition, hence complicating the diagnosis of a mental disorder. This is evident in the case of diagnosing depression among elderly, where the similarity of symptoms of depression to those of dementia, or the presence of a medical condition may override the symptoms of depression [9]. A mental health literacy study among psychiatrically and generally trained nurses employed in a psychiatric hospital has also revealed that despite the training received, the depression vignette was commonly defined as “stress” instead of a psychiatric diagnosis [10]. Those in the field of medicine or nursing have since realized the importance of mental health literacy and have embarked on more proactive approaches in educating and training healthcare students in the area of psychiatry and mental health [11].

Mental health has been traditionally believed to exist on a continuum, with mental well-being at one end of the continuum and mental illness at the other end. Evidence has shown that one can suffer from symptoms of mental illness on a continuum [12, 13]. In other words, while a proportion of the general population might not have met the criteria for a full blown mental disorder, they would have experienced one or more symptoms associated with mental disorders during their lifetime. Studies have also found the belief in a continuum of symptom experience to be associated with less stigmatizing attitudes towards those with mental illnesses [14, 15]. Stigmatization of mental illness has been conceptualized as a process of multiple, interrelated steps and central to this, it is the separation between “us” and “them” that leads to the negative emotional reactions and eventually, discrimination and devaluation of the person with mental illness [16]. Individuals who endorsed stronger continuum belief or agreed that mental health problems are also experienced by many, sometimes are less likely to perceive this differentness between those with and without mental illnesses. In line with this notion, stronger continuum belief could also possibly cloud one’s likelihood to diagnose someone who develops signs and symptoms of mental health problems as having a clinically significant mental disorder. Therefore, we hypothesized that stronger continuum belief would be linked to the non-identification of mental illness in our study.

The current study therefore aimed to examine: (1) the correct recognition rates and continuum beliefs of psychiatric symptoms across five diagnostic conditions namely dementia, alcohol abuse, obsessive compulsive disorder (OCD), schizophrenia and depression, (2) the correlates of correct recognition, (3) the correlates of continuum belief and finally, (4) the relationship between continuum belief and non-identification of mental illness in a nursing student population in Singapore.

## Methods

### Study participants

Students enrolled in four nursing institutions in Singapore were invited to take part in a web-based survey via school email or verbal dissemination by staff personnel during class. A total of 500 students were recruited from the period of April to July 2016. Quota limits were set to ensure adequate participation of students from each of the institutions and across their academic years. Implicit informed consent was obtained. The study was approved by the ethics committee of the relevant institutional review board. Participants received a Starbucks card with monetary value of $20 upon completion of the study.

### Questionnaire

Sociodemographic data (age, gender, ethnicity, household income, current education, professional year) and other information pertaining to the mode of their training in psychiatric nursing (e.g., lecture and small group teaching, clinical placement) were collected using a structured questionnaire. A vignette based approach modeled on the Depression Literacy Questionnaire by Jorm et al. (1997) [5] was adopted to determine the level of mental health literacy among the target study population. Participants were presented with a vignette describing an individual “X” with a specific disorder before completing subsequent questions. They were asked to indicate if they feel the symptoms described in the given vignette were a cause for concern or worry, followed by responding to an open-ended question on what they think X is suffering from. Next, participants were further asked to rate their agreeableness with the following statement: “Sometimes we all behave like X. It is just a question of how severe or obvious this condition is” on a 5-point Likert-scale from (1) strongly disagree to (5) strongly agree. A higher score indicates stronger belief in a continuum of symptom experience. Lastly, they were asked if anyone in their family or close circle of friends ever had problems similar to X and if they had any experience (e.g., volunteering, training) in dealing with a person who has a problem like X for the respective vignette they received.

### Vignettes

A total of five vignettes were used for the current study to describe dementia, alcohol abuse, OCD, schizophrenia and depression. These vignettes were developed through consultation with experienced research psychiatrists and approved by a panel of senior clinical psychiatrists to ensure that each disorder satisfied the DSM-IV criteria. Detailed information on the development of these vignettes can be found in a study conducted among the general population in Singapore [17]. Depression, alcohol abuse and OCD were selected as they were identified as the top three most prevalent disorders in the local population [18] while dementia and schizophrenia were included as local data has also made a strong case for their early detection and treatment [19, 20]. To eliminate inter observer variations and biases, the five vignettes were randomly assigned by the computer to the participants such that each participant only received one vignette and each vignette was administered to 100 participants. Chi-squared analyses were run separately to confirm the absence of unequal distribution of vignette allocation across all sociodemographic and education related factors among the participants.

### Coding of open-ended responses

Response was coded as “Correct recognition” if the respondent was able to accurately name the specific condition. In the case of an ambiguous response, two of the authors (ES and MS) would come to a consensus on how the response would be coded. “Disorder specific symptoms” refers to amnesia, short term memory, confusion for dementia, germophobia, anxiety for OCD, and hallucinations, delusions, paranoia for schizophrenia. “Not an illness” refers to, for example, the lack of self-control, anger management problem, or feelings of insecurity and anxiousness. The response categories reported include only those that have been endorsed by at least 5.0% of the students in a single vignette.

### Power calculation and statistical analyses

Based on a previous study looking at the mental health literacy conducted in the general population in Singapore [17], the overall correct recognition rate of specific conditions was 43.7% using single proportion formula with precision set at 5%. A sample size of 378 participants would therefore be required to ensure adequate power for the study. In all, 500 responses were received from all the institutions and included for further analysis. Statistical analyses were performed using IBM SPSS, version 23. Descriptive statistics were tabulated for the overall sample. Frequency and percentage were calculated for categorical variables, while mean and standard deviation were calculated for all other continuous variables. Multiple logistic regression was performed to determine correlates of correct recognition of mental disorders while multiple linear regression was conducted to examine the correlates of continuum belief (belief that problem is also experienced by others) in our sample. We have also examined the relationship between continuum belief and the non-identification of mental illness after adjusting for covariates using multiple logistic regression. Statistical significance was set at *p* < 0.05 level.

## Results

The characteristics of the sample are displayed in Table 1. The majority of the students were in the younger age group (<21 years old), females, Chinese and had a monthly household income of $4000 and above. A large proportion of students undertaking diploma certification were recruited as they represent the bulk of the nursing student population in Singapore. Most of them had completed their psychiatric nursing training through lectures and small group teaching (79.8%), as well as clinical placement (60.6%). 25.0% had an experience dealing (e.g., through volunteering, training) with someone like the person they had read about in their assigned vignette while 34.8% of them had friends or family members with problems similar to person “X”.

**Table 1:** Profile of study participants (*N* = 500)

		*n*	%
Age group	<21 years	334	66.8
	21 years and above	166	33.2
Gender	Male	83	16.6
	Female	417	83.4
Ethnicity	Chinese	287	57.4
	Malay	134	26.8
	Indian and Others	79	15.8
Monthly household income($)	Below 2000	158	31.6
	2000–3999	160	32.0
	4000 and above	182	36.4
Education	Vocational	100	20.0
	Diploma	300	60.0
	Tertiary	100	20.0
Stage of nursing education (years spent in nursing education)	1^st^ year	147	29.4
	2^nd^ year	166	33.2
	3^rd^ year	102	20.4
	4^th^ year	63	12.6
	5^th^ year	22	4.4
Lecture and small group teaching	Attended	399	79.8
	Not attended	101	20.2
Clinical placement	Attended	303	60.6
	Not attended	197	39.4
Has experience dealing with someone having problems similar to “X”	Yes	125	25.0
	No	375	75.0
Has friends and family with problems similar to “X”	Yes	174	34.8
	No	326	65.2

Table 2 shows the percentage of students mentioning each category to describe the problem in the vignette assigned to them. The correct recognition rates across the five vignettes- dementia, alcohol abuse, OCD, schizophrenia and depression were 77.0%, 58.0%, 86.0%, 46.0% and 85.0% respectively. ‘Depression’ was the term used most often to describe the other four vignettes (apart from their correct recognition and disorder specific symptoms), with 23.0% describing “alcohol abuse” as such. The person X in the “dementia” and “alcohol abuse” vignette were described as suffering from a medical problem by 1.0% and 3.0% of their respective sample and these were rated under irrelevant responses.

**Table 2:** Problem description by the nursing students based on vignette assigned, *n* (%)

	Overall (*N* = 500)	Dementia (*n* = 100)	Alcohol abuse (*n* = 100)	OCD (*n* = 100)	Schizophrenia (*n* = 100)	Depression (*n* = 100)
Recognition						
1. Correct recognition	352 (70.4)	77 (77.0)	58 (58.0)	86 (86.0)	46 (46.0)	85 (85.0)
2. Disorder specific symptoms	33 (6.6)	8 (8.0)	-	6 (6.0)	17 (17.0)	2 (2.0)
3. Other mental disorder- any anxiety disorder	11 (2.2)	1 (1.0)	4 (4.0)	-	5 (5.0)	1 (1.0)
4. Other mental disorder- depression	43 (8.6)	7 (7.0)	23 (23.0)	4 (4.0)	9 (9.0)	-
5. Other mental disorder- miscellaneous	17 (3.4)	1(1.0)	3 (3.0)	4 (4.0)	7 (7.0)	2 (2.0)
6. Mental illness	12 (2.4)	2 (2.0)	1 (1.0)	-	7 (7.0)	2 (2.0)
7. Psychosocial stress	8 (1.6)	-	2 (2.0)	-	1 (1.0)	5 (5.0)
8. Not an illness	15 (3.0)	1 (1.0)	6 (6.0)		5 (5.0)	3 (3.0)
9. Not sure/irrelevant response	9 (1.8)	3 (3.0)	3 (3.0)	-	3 (3.0)	-
Continuum Belief- believes problem is also experienced by many, sometimes						
Agree	291 (58.2)	56 (56.0)	58 (58.0)	61 (61.0)	46 (46.0)	70 (70.0)
Disagree	106 (21.2)	19 (19.0)	21 (21.0)	20 (20.0)	33 (33.0)	13 (13.0)
Undecided	103 (20.6)	25 (25.0)	21 (21.0)	19 (19.0)	21 (21.0)	17 (17.0)
Mean score (S.D.)	3.44 (1.08)	3.45 (0.99)	3.41 (1.08)	3.58 (1.17)	3.08 (1.12)	3.69 (0.95)

Table 3 shows the correlates of correct recognition in the sample. Those of the Malay ethnicity were less likely to correctly identify the mental disorder compared to the Chinese. Nursing school factors such as the years spent in nursing education or the training in psychiatric nursing (e.g., the attendance of clinical placement, lecture and small group teaching) were not significantly associated with the correct recognition of mental disorders. All four vignettes- depression, dementia, OCD and alcohol abuse had higher odds of being correctly recognized compared to the schizophrenia vignette. Those having experience dealing with someone who had a problem similar to the assigned vignette were also more likely to correctly recognize the mental disorder compared to their counterparts.

**Table 3:** Correlates of correct recognition of mental disorders (*N* = 500)

		OR	95% CI		*p*-value
Age group	<21 years	Ref.	-	-	-
	21 years and above	1.424	0.783	2.590	0.247
Gender	Female	Ref.	-	-	-
	Male	0.636	0.358	1.130	0.123
Ethnicity	Chinese	Ref.	-	-	-
	Malay	0.422	0.242	0.738	0.002
	Indian and Others	0.550	0.290	1.044	0.068
Monthly household income	Below 2000	Ref.	-	-	-
	2000–3999	0.899	0.517	1.561	0.705
	4000 and above	0.622	0.357	1.082	0.093
Education	Vocational	Ref.	-	-	-
	Diploma	0.813	0.427	1.548	0.528
	Tertiary	1.622	0.665	3.954	0.287
Stage of nursing education	1^st^ year	Ref.	-	-	-
	2^nd^ year	1.251	0.634	2.467	0.518
	3^rd^ year	2.224	0.877	5.637	0.092
	4^th^ year	1.045	0.408	2.680	0.926
	5^th^ year	1.242	0.316	4.889	0.756
Lecture and small group teaching	Attended	Ref.	-	-	-
	Not attended	1.511	0.781	2.926	0.220
Clinical placement	Attended	Ref.	-	-	-
	Not attended	0.971	0.489	1.930	0.933
Vignette type	Schizophrenia	Ref.	-	-	-
	Depression	8.427	3.985	17.822	<0.001
	Dementia	4.587	2.342	8.982	<0.001
	OCD	9.610	4.576	20.179	<0.001
	Alcohol abuse	1.845	1.002	3.396	0.049
Has experience dealing with someone having problems similar to “X”	No	Ref.	-	-	-
	Yes	1.868	1.126	3.097	0.015
Has friends and family with problems similar to “X”	No	Ref.	-	-	-
	Yes	1.477	0.837	2.605	0.178

*CI* confidence interval, *OR* odds ratio

Table 4 presents the correlates of continuum belief of mental disorders. Our findings revealed that students in the 2^nd^ and 4^th^ year of their nursing education were less likely to endorse a belief in a continuum of symptom experience compared to 1^st^ year students. Those who received the depression and OCD vignettes were morely likely to perceive a continuum of symptom experienced than those who received the schizophrenia vignette. In addition, those who had close friends and family members with problems similar to “X” were more likely to have stronger continnum belief than those without.

**Table 4:** Correlates of continuum belief of mental disorders (*N* = 500)

		β	95% CI		*p*-value
Age group	<21 years	Ref.	-	-	-
	21 years and above	−0.090	−0.337	0.158	0.476
Gender	Female	Ref.	-	-	-
	Male	−0.005	−0.257	0.246	0.966
Ethnicity	Chinese	Ref.	-	-	-
	Malay	0.009	−0.230	0.248	0.940
	Indian and Others	−0.054	−0.331	0.222	0.700
Monthly household income	Below 2000	Ref.	-	-	-
	2000–3999	0.039	−0.194	0.272	0.743
	4000 and above	−0.073	−0.303	0.158	0.535
Education	Vocational	Ref.	-	-	-
	Diploma	0.161	−0.125	0.448	0.269
	Tertiary	0.059	−0.307	0.426	0.750
Stage of nursing education	1^st^ year	Ref.	-	-	-
	2^nd^ year	−0.299	−0.595	−0.003	0.047
	3^rd^ year	−0.061	−0.458	0.336	0.763
	4^th^ year	−0.431	−0.832	−0.030	0.035
	5^th^ year	0.227	−0.372	0.826	0.457
Lecture and small group teaching	Attended	Ref.	-	-	-
	Not attended	−0.103	−0.376	0.169	0.456
Clinical placement	Attended	Ref.	-	-	-
	Not attended	0.129	−0.167	0.426	0.393
Vignette type	Schizophrenia	Ref.	-	-	-
	Depression	0.415	0.116	0.714	0.007
	Dementia	0.234	−0.064	0.532	0.123
	OCD	0.449	0.156	0.742	0.003
	Alcohol abuse	0.262	−0.032	0.557	0.081
Has experience dealing with someone having problems similar to “X”	No	Ref.	-	-	-
	Yes	−0.133	−0.339	0.072	0.202
Has friends and family with problems similar to “X”	No	Ref.	-	-	-
	Yes	0.413	0.189	0.638	<0.001

*CI* confidence interval, *β* beta coefficient

A total of 65 participants (13.0%) in the entire sample did not identify the condition in the vignette as a mental illness and these include those in the categories of “disorder specific symptoms”, “psychosocial stress”, “not an illness”, and “not sure/irrelevant response”. After controlling for all confounding variables, we found higher continuum belief score to be significantly associated with the identification of the condition in the assigned vignette as a non- mental disorder (Table 5). Compared to all other vignettes*—*depression, dementia, OCD and alcohol abuse*—*schizophrenia was also less likely to be recognized as a mental disorder.

**Table 5:** Relationship between continuum belief and the non-identification of mental illness

		OR	95% CI		*p*-value
Continuum belief score		1.360	1.011	1.829	0.042
Age group	<21 years	Ref.	-	-	-
	21 years and above	0.686	0.309	1.523	0.354
Gender	Female	Ref.	-	-	-
	Male	1.899	0.938	3.842	0.075
Ethnicity	Chinese	Ref.	-	-	-
	Malay	1.830	0.907	3.692	0.092
	Indian and Others	1.850	0.813	4.213	0.143
Monthly household income	Below 2000	Ref.	-	-	-
	2000–3999	0.541	0.256	1.143	0.108
	4000 and above	1.176	0.605	2.285	0.663
Education	Vocational	Ref.	-	-	-
	Diploma	1.252	0.566	2.768	0.578
	Tertiary	1.047	0.340	3.227	0.936
Stage of nursing education^a^	1^st^ year	Ref.	-	-	-
	2^nd^ year	0.882	0.389	1.999	0.764
	3^rd^ year	0.456	0.142	1.469	0.188
	4^th^ year	1.129	0.358	3.560	0.837
Lecture and small group teaching	Attended	Ref.	-	-	-
	Not attended	0.677	0.303	1.513	0.342
Clinical placement	Attended	Ref.	-	-	-
	Not attended	1.512	0.655	3.492	0.333
Vignette type	Schizophrenia	Ref.	-	-	-
	Depression	0.266	0.111	0.636	0.003
	Dementia	0.346	0.152	0.790	0.012
	OCD	0.133	0.049	0.363	<0.001
	Alcohol abuse	0.289	0.126	0.664	0.003
Has experience dealing with someone having problems similar to “X”	No	Ref.	-	-	-
	Yes	0.710	0.367	1.373	0.309
Has friends and family with problems similar to “X”	No	Ref.	-	-	-
	Yes	0.535	0.247	1.160	0.113

^a^OR was not estimated for 5^th^ year students (*n* = 22) due to zero case endorsing non-mental illness

All variables except continuum belief score were entered into the logistic regression model as covariates

*CI* confidence interval, *OR* odds ratio

## Discussion

The current study seeks to explore the recognition and perception of mental disorders within the nursing student population in Singapore via a vignette approach. Comparison was made across five diagnoses namely depression, dementia, OCD, alcohol abuse and schizophrenia, which were selected for reasons such as their prevalence and clinical impact within the local general population.

### Correct recognition of mental disorders

Expectedly, our study revealed higher correct recognition rates across most diagnoses among the nursing students compared to the general population in Singapore who were assigned the same vignettes in another study [17]. The greatest difference was reported in the vignette for OCD (86.0% vs 28.7%), followed by schizophrenia (46.0% vs 11.5%), depression (85.0% vs 55.2%), and dementia (77.0% vs 66.3%). The recognition rates for alcohol abuse were almost similar (58.0% vs 57.1%).

The recognition for schizophrenia was the lowest among the five mental disorders in the nursing population with less than 50% of them being able to correctly recognise it. While the recognition for schizohprenia was also the lowest in the general population compared to the other four diagnoses [17], trained nurses at a psychiatric hospital were able to more accurately diagnose schizophrenia compared to depression and mania [10]. Near to a fifth of the students were able to identify at least one of the symptoms such as hallucination or delusion presented in the entire vignette but did not recognise X as suffering from schizophrenia nor a mental illness. Despite being the vignette associated with the lowest continnum belief score (i.e., we do not tend to behave like X), students also did not seem to recognize schizophrenia as a mental disorder compared to the other four vignettes contrary to our hypothesis. This may suggest a critical lack of understanding for schizophrenia as a mental disorder in this group despite the training in psychiatric nursing received.

It was noteworthy, that the nursing students did not perform much better in term of their recognition for the alcohol abuse vignette. Almost a quarter of the students who received the vignette on alcohol abuse/dependence misidentified the excessive alcohol drinking as depression even though there was no indication of any mood symptom within the vignette passage. These students might have possibly looked beyond the presenting symptoms and perceived depression as the the underlying cause for the alcohol abuse. In other words, the drinking behavior was deemed to be a sign of depression, instead of being a disorder itself. In addition, the misidentification could be influenced by their knowledge of the high comorbidity and strong association between alcohol use disorder and depression based on past research studies [21–23]. While the identification of either depression or alcohol use disorder should lead to further inquiry into the symptoms of the other in clinical practice, the problem of misdiagnosis may impede the formulation and delivery of effective treatment for individuals with alcohol use disorder. This can have significant potential implications for healthcare providers.

Our analysis also revealed that students of the Malay ethnicity were less likely to correctly identify the mental disorders compared to the students of Chinese ethnicity. Again, this phenomenon has also been reflected in the general population where the Malays and the Indians were more likely to describe certain disorders such as dementia as “not a disorder” or use disorder specific symptoms [17]. One possibility suggested was that these ethnic groups may have normalized or attributed the manifestations of certain mental disorders to reasons other than a disease state. Data in our sample also suggests that Malay students tend not to recognise the vignettes as mental disorders compared to the Chinese although this is not significant (*p* = 0.092). Nevertheless, more research would need to be done to explore the underlying causes or tendencies for such ethnic differences in the recognition of mental problems.

### Continuum belief of mental disorders and its association with mental illness identification

While comparing continuum belief across the five mental diagnoses, we found the percentage of those who believe in a continuum of symptoms to be the lowest for schizophrenia compared to the other conditions- schizophrenia (46.0%) followed by dementia (56.0%), alcohol abuse (58.0%), OCD (61.0%), and depression (70.0%). Compared to the data from the general population in Singapore, the finding is almost similar in terms of their order (except dementia) but not the magnitude: schizophrenia (32.7%), alcohol abuse (35.6%), OCD (36.8%), dementia (46.8%) and depression (57.9%) [24]. Other studies have also found a lower agreement or percentage of respondents who belived in a symptom continuity for schizophrenia compared to depression [14, 15, 25]. The symptom continuity score for alcohol abuse was the next lowest after schizophrenia. The percentages of respondents who agreed with symptom continuity were found to be similar for schizophrenia (26%) and alcohol abuse (27%) in one study [14]. It is noteworthy that a higher proportion of students agreed with symptom continuum (70% for depression and 46% for schizophrenia) compared to those in the populations surveys including that of Singapore’s (42% and 26% respectively in Germany; 58.2% and 28.5% respectively in France) [14, 24, 25]. One possible reason might be that nursing students have more opportunities for exposure to individuals with mental illnesses during their course of study, hence resulting in higher endorsement of symptom continuum compared to the general population.

Our data also suggested that among nursing students who have close friends and family with problems similar to “X” (but not those who have experience dealing with someone like “X”), were more likely to endorse greater continuum belief than their counterparts. Again, this result may not be surprising given that long term exposure may play an important role in shaping beliefs and attitudes. Prolonged and intimate exposure has been suggested to lead to a positive attitudinal or perceptual change towards mental illness with improved benevolence (the view that the person is suffering psychologically, due to their misfortune, should be the subject of a kind and paternalistic protectionism based on care, personal attention, and material comfort), mental hygiene ideology (the idea that the person suffering from mental illness is similar to normal people, but with quantitative rather than qualitative differences) and interpersonal aetiology (the belief that the real cause of a mental illness are the problematic interpersonal relations) [26, 27]. Individuals with mental illness are often recognized as “strangers” and will remain a stranger when there is a lack of available cognitive patterns of interpretation. However, personal contact with affected individuals allows one to gain better awareness on the causes, symptoms and treatment options of mental disorders and thus provides one with the interpretational patterns to eliminate the recognized strangeness from mentally ill patients and eventually making him or her, an accepted other member [28]. On the other hand, our study found a negative association between years spent in nursing education and continuum belief score. The relationship between years of nursing education and continuum belief has not been well-researched and it is unknown why more experienced nursing students are less likely to endorse symptom continuity for mental disorders. Further research is needed to understand this.

Our study may be the first to examine the association between continuum belief and the identification of mental illness. Our study revealed higher continuum belief score to be associated with the identification of person “X” as not having a mental disorder or vice versa after controlling for possible covariates. Studies looking at the relationship with stigmatization used the belief in continuity of mental illness symptoms as a proxy for separation between “us” and “them” proposed that those who endorsed stronger belief tend to show reduced stigma and less negative atittudes towards individuals with mental illness [14, 15, 25, 29]. Our study suggested that those who endorsed strong continuum belief may not even identify those individuals experiencing the given mental health symptoms as having a mental disorder, and instead may perceive it as the extreme end of symptoms perceived by the population in general, leading to lower stigmatizing attitudes and higher acceptance. Nonetheless, on a negative note, this finding has an important implication as it may suggest that healthcare trainees who endorse strong continuum belief are less likely to see mental health symptoms experienced by one to be a manifestation of a mental disorder, and are hence, unlikely to refer these individuals for psychiatric treatment.

The strengths of the study include that it has recruited a good representative of nursing students from the four main nursing schools in Singapore. The use of similar vignettes as those by Chong et al. (2016) [17] allows us to make meaningful comparison in terms of mental health literacy with the general population. The study also included factors such as intra-nursing school training in psychiatric nursing and continuum beliefs and explored their relationship with mental health literacy. To the best of our knowledge, no other study has looked into these variables in this area. One limitation, however, pertains to the use of vignettes that described persons with prototypical and severe symptoms, may not reflect real-life cases. Given that this is a group of healthcare professionals-to-be, it may be crucial to explore their ability to recognize more complicated cases such as those with comorbidities, and those with prodromal or uncommon symptoms.

## Conclusion

The current study highlighted the need for further training and education to raise mental health literacy among the nursing student population, particularly in schizophrenia and alcohol abuse although this group performed better in terms of recognition across all five mental diagnoses compared to the general population. Our study also revealed that slightly more than 50% of the students agreed in a symptom continuum from mental health to mental illness. Nursing schools and placement institutions may have to play an important role to ensure that their students are mindful of such continuum belief that could possibly influence their recognition for mental disorders.
